# Validation of Nine Different Prognostic Grading Indexes for Radiosurgery of Brain Metastases in Breast Cancer Patients and Development of an All-Encompassing Prognostic Tool

**DOI:** 10.3389/fonc.2020.01557

**Published:** 2020-09-02

**Authors:** Fabian Weykamp, Rami A. El Shafie, Laila König, Katharina Seidensaal, Tobias Forster, Nathalie Arians, Sebastian Regnery, Philipp Hoegen, Thomas M. Deutsch, Andreas Schneeweiss, Jürgen Debus, Juliane Hörner-Rieber

**Affiliations:** ^1^Department of Radiation Oncology, Heidelberg University Hospital, Heidelberg, Germany; ^2^Heidelberg Institute of Radiation Oncology (HIRO), Heidelberg, Germany; ^3^Department of Obstetrics and Gynecology, Heidelberg University Hospital, Heidelberg, Germany; ^4^National Center for Tumor Diseases (NCT), Heidelberg, Germany; ^5^Department of Radiation Oncology, Heidelberg Ion-Beam Therapy Center (HIT), Heidelberg University Hospital, Heidelberg, Germany; ^6^Clinical Cooperation Unit Radiation Oncology, German Cancer Research Center (DKFZ), Heidelberg, Germany; ^7^German Cancer Consortium (DKTK), Partner Site Heidelberg, Heidelberg, Germany

**Keywords:** stereotactic radiosurgery, breast cancer, brain metastases, prognostic tool, breast graded prognostic assessment, prognostic grading index, overall survival

## Abstract

**Purpose:** Several prognostic indexes for overall survival (OS) after radiotherapy of brain metastases in breast cancer patients exist but are mainly validated for whole-brain radiotherapy or not specifically for breast cancer patients. To date, no such index provides information beyond mere OS.

**Methods:** We retrospectively analyzed 95 breast cancer patients treated with stereotactic radiosurgery for 203 brain metastases. The Kaplan–Meier method with log-rank test was used to assess OS, local control (LC), distant cranial control (DCC), and extracranial control (EC). Cox regression was applied to detect prognostic outcome factors. A point scoring system was designed to stratify patients based on outcome. Nine established prognostic indexes were analyzed using the concordance index (c-index).

**Results:** Two out of nine analyzed prognostic indexes for OS showed a significant c-index, the breast graded prognostic assessment (bGPA; 0.631; 95% CI, 0.514–0.748; *p* = 0.037) and the modified bGPA (mod-bGPA; 0.662; 95% CI, 0.547–0.777; *p* = 0.010). Significant results from multivariate analysis (Karnofsky Performance Score, Her2/neu receptor status, extracranial control) were used to generate a new point system: the breast cancer stereotactic radiotherapy score (bSRS), which discriminated three significantly different prognostic groups, for LC, DCC, EC, and OS, respectively. However, the c-index was only significant for OS (0.689; 95% CI, 0.577–0.802; *p* = 0.003).

**Conclusions:** The new bSRS score was superior to the bGPA and mod-bGPA scores for prognosis of OS. The bSRS is easy to use and the first tool, which might also provide outcome assessment beyond mere OS. Future studies need to validate these findings.

## Background and Purpose

In autopsy studies, central nervous system metastases were detected in up to 30% of breast cancer patients (1). Brain metastases represent a major limiting survival factor for breast cancer patients (2). Stereotactic radiosurgery (SRS) is the recommended treatment for limited brain metastases (3–6).

One of the oldest scoring tools to solely estimate overall survival (OS) in patients with brain metastases is the Graded Prognostic Assessment (GPA) index, which consists of age, Karnofsky performance status (KPS), number of brain metastases, and presence of extracranial metastases. This prognostic tool was developed through analysis of several RTOG studies (7–11). All of the mentioned studies mainly took place in the pre-Her2/neu receptor status era, only a small proportion of breast cancer patients were included, and no patient received SRS without concomitant whole-brain radiotherapy (WBRT). Sperduto et al. then developed the breast GPA (bGPA) for OS prediction in the year 2012, which specifically addressed breast cancer patients. This score includes age, KPS, and Her2/neu as well as hormone receptor status. The respective validation study comprised 400 breast cancer patients, 115 of which received SRS only. Interestingly, unlike the original GPA, the number of brain metastases and whether extracranial metastases were present were left out in the scoring system. Subsequently, Subbiah et al. developed a modified bGPA (mod-bGPA) for OS assessment by reintegrating the number of brain metastases into the scoring system. The respective study cohort comprised 1,552 breast cancer patients; however, only 164 of which (11%) received SRS alone (5). Furthermore, Sperduto et al. updated their bGPA (u-bGPA) and reintegrated the number of brain metastases and the presence of extracranial metastases (12). Table 1 illustrates an overview including several additional prognostic tools.

**Table 1:** Overview of prognostic scoring indexes for radiotherapy in brain metastases (x marks included factors).

	**Hormone or Her2/neu receptor status**	**Karnofsky performance score**	**Extracranial metastases present**	**Extracranial progression**	**Age**	**Volume of largest brain metastasis**	**Primary tumor controlled**	**Number of brain metastases**
Recursive partitioning analysis (13)	–	x	x	–	x	–	x	–
Modified recursive partitioning analysis (14)	–	x	–	–	x	–	–	–
Point scoring system (15)	x	–	–	x	–	–	–	x
Graded prognostic assessment (3)	–	x	x	–	x	–	–	x
Breast graded prognostic assessment (4)	x	x	–	–	x	–	–	–
Modified breast graded prognostic assessment (5)	x	x	–	–	x	–	–	x
Updated breast graded prognostic assessment (12)	x	x	x	–	x	–	–	x
Score index for radiosurgery in brain metastases (16)	–	x	x	–	x	x	–	x
Basic score for brain metastases (17)	–	x	x	–	–	–	x	–
Breast cancer stereotactic radiotherapy score (present study)	x	x	–	x	.	–	–	–

Recent studies investigated prognostic factors for OS in breast cancer patients treated with SRS only. However, these studies did not analyze or compare the prognostic validity of the scoring tools in this particular setting. Instead, only a plane description of the number of patients in the respective prognostic group was performed (6, 18).

To our best knowledge, there is no available prognostic tool for breast cancer patients with brain metastases, which exceeds the assessment of mere OS. However, it is of high importance to also estimate local control (LC), distant cranial control (DCC), and extracranial control (EC) when discussing different treatment options and their optimal timing in an interdisciplinary tumor board, including SRS, WBRT, and intensification of systemic therapy.

On top of validating different OS scoring tools for SRS in cerebral metastasized breast cancer patients, we therefore sought to develop a new score, which comprises prognostic assessment of LC, DCC, EC, and OS altogether.

## Methods

### Patients and Treatment Characteristics

We retrospectively analyzed female breast cancer patients treated with SRS for brain metastases in the Department of Radiation Oncology at Heidelberg University Hospital from 05/2005 to 10/2019. Until 2016, patients were treated with a conventional linear accelerator (Siemens Mevatron, Erlangen, Germany, or Elekta Versa HD, Stockholm, Sweden), while from 2016 on, the CyberKnife M6 (Accuray Inc., Sunnyvale California, USA) was primarily used for radiosurgery.

All patients received an individual scotch cast or thermoplastic mask for head fixation. A computed tomography (CT) scan as well as a magnetic resonance imaging (MRI) scan of the head was performed for treatment planning. The gross tumor volume (GTV) of each brain metastasis was delineated on both contrast-enhanced CT and MRI. The addition of a safety margin (1–3 mm, isotropic) resulted in the planning target volume (PTV). For conventional linac-based radiosurgery, a PTV margin of 3 mm was applied to account for geometry uncertainty, while for robotic radiosurgery, a PTV margin of only 1 mm was used. The radiation dose was prescribed according to current German (19) and European (20) guidelines, depending on the size of the respective metastasis. Most patients received 20 or 18 Gy, prescribed to the 70% isodose and covering at least 98% of the PTV.

The biologically effective dose (BED) was calculated using the linear–quadratic model (21). An α/β ratio of 10 was assumed for brain metastases.

$$\begin{array}{l} BED\;\left(Gy\right)=single\;dose\\ \times \;number\;of\;fractions\;(1+\;\frac{single\;dose}{\alpha /\beta }) \end{array}$$

Ethics approval for the study and a waiver of written informed consent were granted by the Heidelberg University ethics committee on April 12, 2018 (#S-172/2018). Patient confidentiality was maintained by anonymizing patient data to remove any identifying information.

### Endpoints and Statistical Methods

LC, DCC, EC, and OS were calculated starting from the last day of SRS. In this study, LC refers to the high-dose area surrounding the respective irradiated metastasis. Recurrences in the brain outside the high-dose area were defined as distant cranial failure. LC was calculated for each individual lesion. DCC, EC, and OS were calculated per patient. Toxicity was evaluated using the Common Terminology Criteria for Adverse Events (CTCAE v. 5.0).

The first follow-up MRI scan of the head was performed 6–8 weeks after completion of the SRS. Further follow-up was done according to German guidelines and regularly included a contrast-enhanced MRI scan of the head every 3 months.

LC, DCC, EC, and OS were estimated using the Kaplan–Meier method. Survival curves were compared applying the log-rank test. Cox regression was used for univariate analysis. Multivariate cox models were calculated including all variables with *p* ≤ 0.1 from univariate analysis.

The prognostic value of the nine investigated different prognostic scoring tools, shown in Table 1, was assessed by the concordance index (c-index), where a value of 1.0 represents a perfect prognostic scoring tool. A *p* < 0.05 was considered statistically significant.

Results from multivariate analysis (Table 2) were used to generate a new, all-encompassing index, which we refer to as the breast cancer stereotactic radiotherapy score (bSRS; Table 3). It encompasses the Her2/neu receptor status (independently significant for OS), the KPS (independently significant for OS and DCC), and the diagnosis of extracranial progression prior to SRS (borderline significant for OS and independently significant for LC and EC). We then attributed respective points proportionally to their hazard ratio in multivariate analysis (Table 2), with a higher score representing a more favorable outcome in terms of LC, DCC, EC, and OS. A positive Her2/neu receptor status (HR 0.8) resulted in 1 point, reflecting the independently better OS. A KPS of at least 80% (HR 0.4) led to 2 points accordingly, representing twice the effect of the Her2/neu receptor status. However, since the bSRS was designed as a multidimensional tool, we needed to modify the scoring process. Optimal results for LC, DCC, EC, and OS altogether were obtained, if the aforementioned 2 points were attributed only to patients with a KPS of at least 90% and 1 point in case of a KPS of 80%. The same accounted for patients with extracranial progression in terms of LC (HR 4.8). Best results were obtained for all four study endpoints, if 4 points were attributed to patients with extracranial control, reflecting a more favorable outcome. Thus, the maximum of seven points was achieved by patients who were in excellent clinical condition (KPS at least 90%), had stable or absent extracranial disease, and had a positive Her2/neu receptor status. Afterward, patients were allocated to three subgroups according to their respective points (0–4, 5, and 6–7), where 0–4 points represent the poor prognosis subgroup and 6–7 points stand for the subgroup with excellent prognosis in terms of LC, DCC, EC, and OS altogether. All statistical analyses were performed with SPSS software (IBM SPSS Version 24.0).

**Table 2:** Multivariate analysis of prognostic factors influencing local control (LC), distant cranial control (DCC), extracranial control (EC), and overall survival (OS).

	**LC**			**DCC**			**EC**			**OS**		
**Factors**	**HR**	**95% CI**	***P***	**HR**	**95% CI**	***p***	**HR**	**95% CI**	***p***	**HR**	**95%-CI**	***p***
Karnofsky Performance Score ≥80%				0.367	[0.150; 0.899]	***0.028***	0.569	[0.248; 1.303]	*0.182*	0.372	[0.197; 0.703]	***0.002***
Hormone receptor positive	0.589	[0.298; 1.164]	*0.128*									
Her2/neu receptor positive										0.795	[0.649; 0.974]	***0.027***
Synchronous metastases				0.642	[0.265; 1.551]	*0.324*						
Time to metastases ≥18 months	0.064	[0.010; 0.413]	***0.004***									
Single brain metastasis	0.375	[0.056; 2.509]	*0.312*	0.582	[0.256; 1.322]	*0.196*						
Solitary brain metastasis							0.576	[0.194; 1.715]	*0.322*	0.784	[0.350; 1.756]	*0.554*
Prior whole-brain radiotherapy	3.497	[0.863; 14.176]	***0.079***									
Irradiated lesion is symptomatic	2.702	[0.030; 7.086]	***0.032***									
Extracranial metastases *n* > 5										1.518	[0.793; 2.909]	*0.208*
Extracerebral progression in last restaging prior to cerebral radiation	4.759	[1.148; 19.730]	***<0.001***				2.872	[1.766; 4.674]	***<0.001***	1.538	[0.949; 2.492]	***0.080***
≥5 additional brain metastases, stable after prior therapy and therefore not irradiated				1.108	[0.226; 5.421]	*0.899*	2.525	[0.812; 7.816]	0.110	1.451	[1.463; 4.545]	*0.523*
BED ≥60 Gy (α/β = 10)	0.306	[0.091; 1.026]	***0.055***									

*BED, biologically effective dose; CI, confidence interval; HR, hazard ratio. For Her2/neu receptor rich, data was missing for 5 patients and 7 patients had missing data on extracranial progression. Bold italics indicates the statistical significance p < 0.1. Bold, italics, and underlined indicates the statistical significance p < 0.05*.

**Table 3:** Breast cancer scoring tool for radiosurgery (bSRS).

**Points**	**0**	**1.0**	**2.0**	**4.0**
Karnofsky score	≤ 70%	80%	90–100%	–
Her2/neu receptor	Negative	Positive	–	–
Extracranial control	No	–	–	Yes

## Results

Median age was 57 years (range 31–83 years) with a median KPS of 80%. Most patients suffered from additional extracranial metastases (62.2%). About a third (31.6%) had already been treated with WBRT for prior brain metastases. At radiosurgery, all patients were diagnosed with a controlled primary tumor. Further patient characteristics are illustrated in Table 4. Table 5 shows characteristics of the SRS treatment. Most patients received treatment of a single lesion (51.6%) with a maximum of seven irradiated brain metastases.

**Table 4:** Patient characteristics (n = 95).

**Median age**	**57 years**	**Range 31–83 years**
Median Karnofsky score	80%	Range 50–100%
Hormone receptor positive	56	58.9%
Her2/neu receptor positive	34	35.8%
TNBC	25	26.3%
Her2/neu receptor status unknown	5	5.3%
Synchronous metastases	16	16.8%
Median time from initial diagnosis to metastases^*^	37 months	Range 1–276 months
Extracranial metastases in total		
n = 0	35	36.8%
n = 1–5	20	21.1%
n > 5	40	41.1%
Prior WBRT	30	31.6%
Median time from WBRT to stereotactic radiation	16 months	Range 1–47 months
Prior resection of brain metastases	17	17.8%
Extracranial progression within 4 weeks before radiosurgery	6	6.3%
Solitary brain metastasis	22	23.2%
Single brain metastasis	37	38.9%
Chemotherapy within 4 weeks before radiation	24	25.3%

* *Excluding patients with synchronous metastases; TNBC, triple-negative breast cancer; WBRT, whole-brain radiotherapy*.

**Table 5:** Treatment characteristics (*n* = 203).

**Number of irradiated brain lesions**		**Range 1–7**
*n* = 1	49	51.6%
*n* = 2–3	35	36.9%
*n* > 3	11	11.7%
Infratentorial involvement	46	21.4%
Median prescribed total dose	20 Gy	Range 12.0–30.0 Gy
Median fractions	1	Range 1–6
Median-dose inhomogeneity	70%	Range 70–80%
Median EQD2 (α/β = 10)	50.0 Gy	Range 18.8–50.8 Gy
Median BED (α/β = 10)	60.0 Gy	Range 22.5–60.9 Gy

*BED, biologically effective dose; EQD2, equivalent dose at 2 Gy*.

Seventeen (17.9%) grade I acute toxicities were described at first follow-up (mainly mild headache, nausea, or dizziness) and 3 (3.2%) grade II toxicities were documented (nausea, cerebral edema, and headache). In six patients, radiation induced blood–brain barrier disruptions were detected, of whom two underwent surgery, which confirmed radionecrosis and no tumor recurrence. Median imaging follow-up was 11.9 months (range 0.8–105.3).

### Local Control

Local control, defined as a stable or regressive contrast enhancement in the T1-weighted MRI, was 90.6% at 12 months and 71.6% at 24 months.

Univariate analysis (Table 6) showed hormone receptor positivity (HR = 0.626, CI [0.407; 0.961], *p* = 0.032), time to metastases of any kind ≥18 months (HR = 0.239, CI [0.090; 0.634], *p* = 0.004), and a BED ≥60 Gy (HR = 0.378, CI [0.160; 0.894], *p* = 0.027) as positive prognostic factors, whereas symptomatic brain lesions were associated with worse LC (HR = 2.702, CI [0.030; 7.086], *p* = 0.043) as well as extracerebral progression within 4 weeks prior to SRS (HR = 5.265, CI [2.534; 10.940], *p* < 0.001). In multivariate analysis, time to metastases ≥18 months (HR = 0.064, CI [0.010; 0.413], *p* = 0.004) was identified as an independent positive prognostic factor, whereas symptomatic brain lesions (HR = 2.702, CI [0.030; 7.086], *p* = 0.032) and extracerebral progression within 4 weeks prior to SRS (HR = 4.759, CI [1.148; 19.730], *p* < 0.001) were found to be negative prognostic factors for LC (Table 2).

**Table 6:** Univariate analysis of prognostic factors influencing local control (LC), distant cranial control (DCC), extracranial control (EC), and overall survival (OS).

	**LC**			**DCC**			**EC**			**OS**		
**Factors**	**HR**	**95%-CI**	***p***	**HR**	**95%-CI**	***p***	**HR**	**95% CI**	***P***	**HR**	**95% CI**	***p***
Age under 60 years	0.682	[0.186; 2.496]	*0.563*	1.151	[0.590; 2.247]	*0.679*	0.980	[0.485; 1.978]	*0.955*	0.698	[0.415; 1.174]	*0.175*
Karnofsky Performance Score ≥80%	0.754	[0.357; 1.592]	*0.459*	0.440	[0.211; 0.916]	***0.028***	0.493	[0.225; 1.081]	***0.077***	0.355	[0.202; 0.625]	***<0.001***
Hormone receptor positive	0.626	[0.407; 0.961]	***0.032***	0.844	[0.616; 1.158]	*0.294*	1.041	[0.724; 1.496]	*0.828*	0.921	[0.709; 1.197]	*0.538*
Her2/neu receptor positive	0.929	[0.753; 1.145]	*0.488*	0.878	[0.703; 1.097]	*0.251*	0.834	[0.652; 1.076]	*0.149*	0.810	[0.669; 0.981]	***0.031***
Synchronous metastases	1.012	[0.479; 2.137]	*0.976*	0.398	[0.153; 1.033]	***0.058***	0.615	[0.237; 1.600]	*0.319*	0.538	[0.243; 1.190]	*0.126*
Time to metastases ≥18 months	0.239	[0.090; 0.634]	***0.004***	0.711	[0.328; 1.539]	*0.386*	0.618	[0.262; 1.457]	*0.271*	1.001	[0.996; 1.007]	*0.715*
Single brain metastasis	1.272	[0.622; 2.603]	***0.098***	0.545	[0.274; 1.082]	***0.083***	0.814	[0.398; 1.665]	*0.572*	0.894	[0.529; 1.510]	*0.675*
Solitary brain metastasis	1.306	[0.548; 3.112]	*0.546*	0.599	[0.268; 1.338]	*0.211*	0.406	[0.142; 1.160]	***0.093***	0.571	[0.297; 1.097]	***0.092***
Prior resected brain metastases	1.236	[0.545; 2.806]	*0.612*	1.430	[0.620; 3.299]	*0.402*	0.038	[0.001; 2.359]	*0.120*	1.144	[0.537; 2.434]	*0.728*
Prior whole-brain radiotherapy	2.086	[0.881; 4.939]	***0.095***	0.993	[0.503; 1.960]	*0.983*	1.114	[0.530; 2.345]	*0.775*	0.908	[0.514; 1.601]	*0.738*
Irradiated lesion or at least one of them = infratentorial	1.612	[0.825; 3.184]	*0.161*	1.608	[0.786; 3.287]	*0.193*	1.275	[0.591; 2.751]	*0.535*	1.444	[0.818; 2.550]	*0.205*
Irradiated lesion = symptomatic	2.702	[0.030; 7.086]	***0.043***	1.628	[0.791; 3.351]	*0.185*	1.418	[0.645; 3.117]	*0.385*	1.000	[0.561; 1.782]	*1.000*
Extracranial metastases *n* = 0	1.495	[0.777; 2.879]	*0.229*	0.801	[0.418; 1.569]	*0.532*	0.499	[0.216; 1.153]	*0.104*	0.685	[0.396; 1.186]	*0.177*
Extracranial metastases *n* > 5	1.420	[0.774; 2.605]	*0.258*	1.163	[0.619; 2.188]	*0.639*	1.619	[0.818; 3.208]	*0.167*	1.676	[0.999; 2.811]	***0.051***
Extracerebral progression in last restaging prior to cerebral radiation	5.265	[2.534; 10.940]	***<0.001***	0.857	[0.315; 2.331]	*0.762*	2.809	[1.764; 4.474]	***<0.001***	1.676	[1.047; 2.685]	***0.032***
≥5 additional brain metastases, stable after prior therapy and therefore not irradiated	1.424	[0.341; 5.955]	*0.628*	2.870	[0.855; 9.637]	***0.088***	2.833	[0.970; 8.267]	***0.057***	3.705	[1.435; 9.564]	***0.007***
Irradiated cranial metastases *n* ≤ 3	1.464	[0.743; 2.884]	*0.271*	0.827	[0.360; 1.899]	*0.655*	1.569	[0.551; 4.467]	*0.399*	1.800	[0.718; 4.516]	*0.210*
BED ≥60 Gy (α/β = 10)	0.378	[0.160; 0.894]	***0.027***	1.158	[0.616; 2.174]	*0.649*	0.997	[0.499; 1.990]	*0.993*	1.370	[0.796; 2.356]	*0.255*
Longest axial diameter of brain metastasis ≥3.0 cm	2.522	[0.333; 19.076]	0.370	0.676	[0.092; 4.960]	*0.700*	0.046	[0.000; 95.815]	*0.430*	1.050	[0.255; 4.313]	*0.946*

*BED, biologically effective dose; CI, confidence interval; HR, hazard ratio. For Her2/neu receptor rich, data was missing for 5 patients and 7 patients had missing data on extracranial progression. Bold italics indicates the statistical significance p < 0.1. Bold, italics, and underlined indicates the statistical significance p < 0.05*.

### Distant Intracranial Control

Forty out of 95 patients (42.1%) were diagnosed with cerebral progression distant to the SRS lesion during follow-up. One- and two-year DCC rates were 65.3 and 36.7%. KPS (HR = 0.440, CI [0.211; 0.916], *p* = 0.028) was revealed as a significant favorable prognostic factor in univariate analysis (Table 6), with synchronous diagnosis of metastases (HR = 0.398, CI [0.153; 1.033] *p* = 0.058) and single brain metastasis (HR = 0.545, CI [0.274; 1.082], *p* = 0.083) at borderline significance level. If patients had presence of ≥5 additional brain metastases, which were stable form prior therapy and therefore not irradiated, it was found to be a borderline significant negative factor associated with worse DCC (HR = 2.870, CI [0.855; 9.637], *p* = 0.088). After adjusting for potential confounding variables on multivariate analysis, none of the aforementioned factors remained significant (Table 2).

### Extracranial Control

During follow-up, 31 of 88 patients developed extracranial progression (35.2%). Estimated 1- and 2-year EC rates were 57.5 and 46.5%.

Extracerebral progression within 4 weeks prior to SRS (HR = 2.809, CI [1.764; 4.474], *p* < 0.001) was identified as an independent prognostic factor for further extracerebral progression after SRS (Table 2).

### Overall Survival

Sixty patients (62.1%) died during follow-up time. One- and two-year OS were 60.9 and 37.8%, respectively. Univariate analysis revealed a KPS ≥80% (HR = 0.355, CI [0.202; 0.625], *p* < 0.001) and Her2/neu positivity (HR = 0.810, CI [0.669; 0.981], *p* = 0.031) as significant factors for superior OS, with a solitary brain metastasis (HR = 0.571, CI [0.297; 1.097], *p* = 0.092) at borderline significance level (Table 6). Five or more not irradiated brain metastases (HR = 3.705, CI [1.435; 9.564], *p* = 0.007) were significantly associated with inferior OS, with extracranial metastases *n* > 5 (HR = 1.676, CI [0.999; 2.811], *p* = 0.051) and extracerebral progression in last restaging prior to cerebral radiation (HR = 1.676, CI [1.047; 2.685], *p* = 0.032) at borderline significance level. In multivariate analysis, a KPS ≥80% (HR = 0.355, CI [0.202; 0.625], *p* < 0.001) and Her2/neu positivity (HR = 0.810, CI [0.669; 0.974], *p* = 0.027) were independently associated with improved OS (Table 2), with extracranial progression at borderline significance level (HR = 1.538, CI [0.949; 2.492], *p* = 0.080).

### Validation of Nine Different Prognostic Grading Indexes

**Figure 1:**
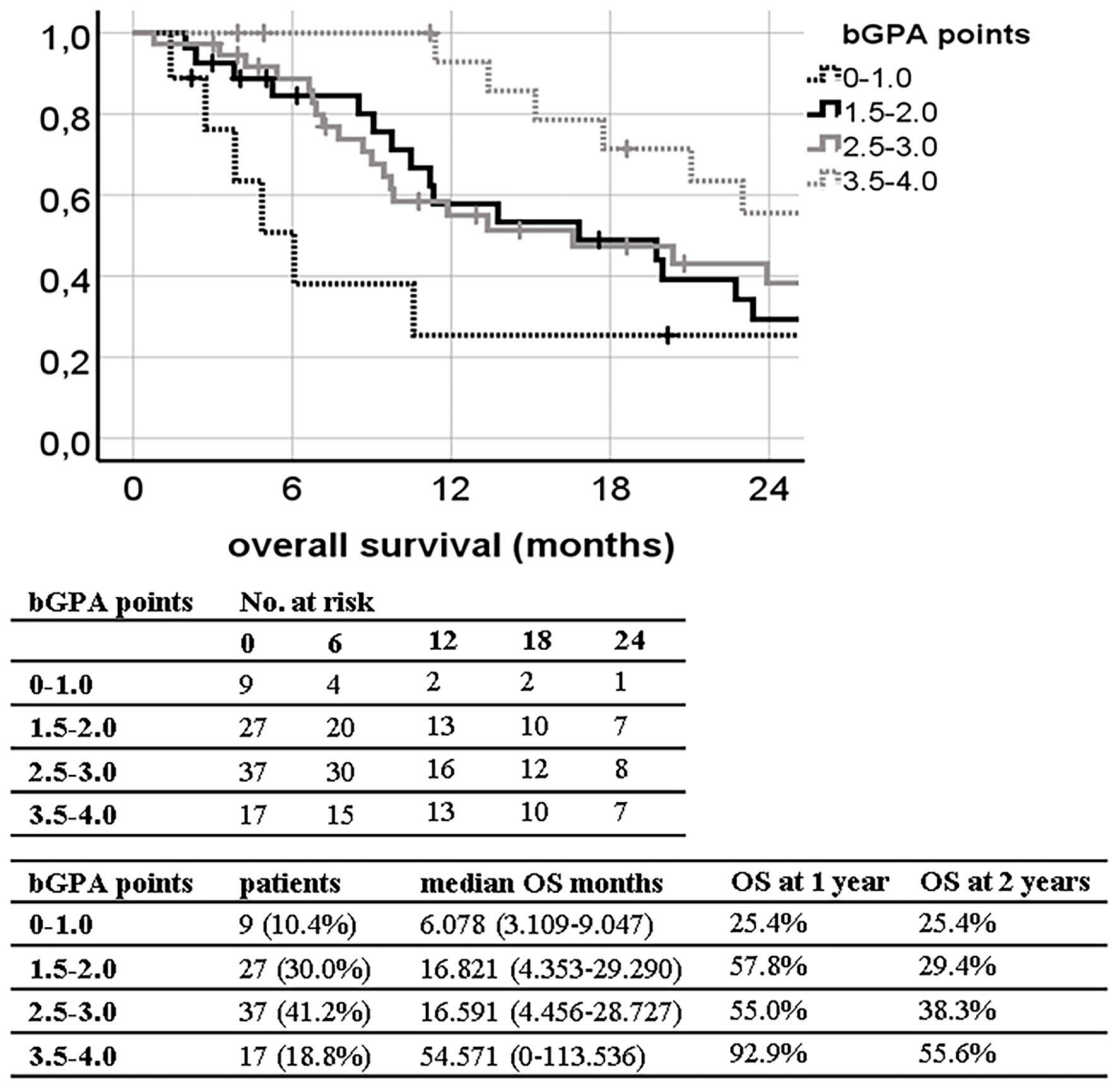
Kaplan–Meier curves for overall survival (OS) divided by the breast graded prognostic assessment (bGPA) in our patient population (log-rank *p* = 0.030; concordance index 0.631; 95% CI, 0.514–0.748; *p* = 0.037) with median OS and 1- and 2-year OS estimates.

**Figure 2:**
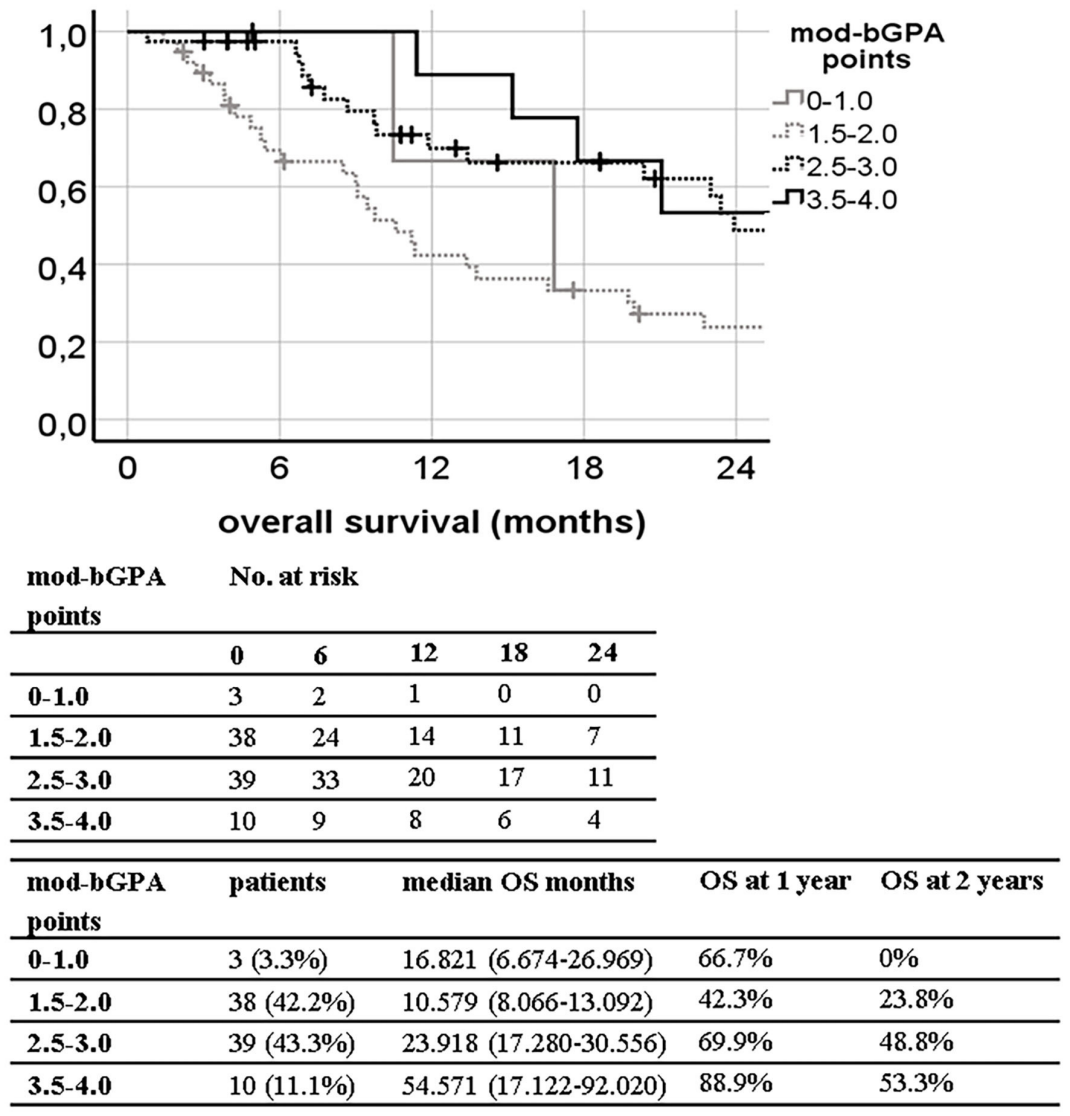
Kaplan–Meier curves for overall survival (OS) divided by the modified breast graded prognostic assessment (mod-bGPA) in our patient population (log-rank *p* = 0.003; concordance index 0.662; 95% CI, 0.547–0.777; *p* = 0.010) with median OS and 1- and 2-year OS estimates.

Two out of nine tested prognostic grading indexes shown in Table 1 provided a reliable ranking of survival groups according to their significant c-index, namely, the bGPA (c-index 0.631; 95% CI, 0.514–0.748; *p* = 0.037) and the mod-bGPA (c-index 0.662; 95% CI, 0.547–0.777; *p* = 0.010). Kaplan–Meier curves, median overall survival, and estimates of 1- and 2-year OS are shown in Figures 1, 2. The other, unsuccessfully tested prognostic scores had insignificant c-indexes, namely, the original GPA (c-index 0.531; 95% CI, 0.407–0.655; *p* = 0.615), the u-bGPA (c-index 0.583; 95% CI, 0.460–0.705; *p* = 0.188), the BSBM (c-index 0.579; 95% CI, 0.462–0.696; *p* = 0.197), the RPA (c-index 0.470; 95% CI, 0.351–0.590; *p* = 0.629), the SIR (c-index 0.566; 95% CI, 0.447–0.686; *p* = 0.279), the PSS (c-index 0.620; 95% CI, 0.502–0.793; *p* = 0.055), and the mRPA (c-index 0.512; 95% CI, 0.394–0.631; *p* = 0.845).

### Breast Cancer Stereotactic Radiotherapy Score (bSRS)

**Figure 3:**
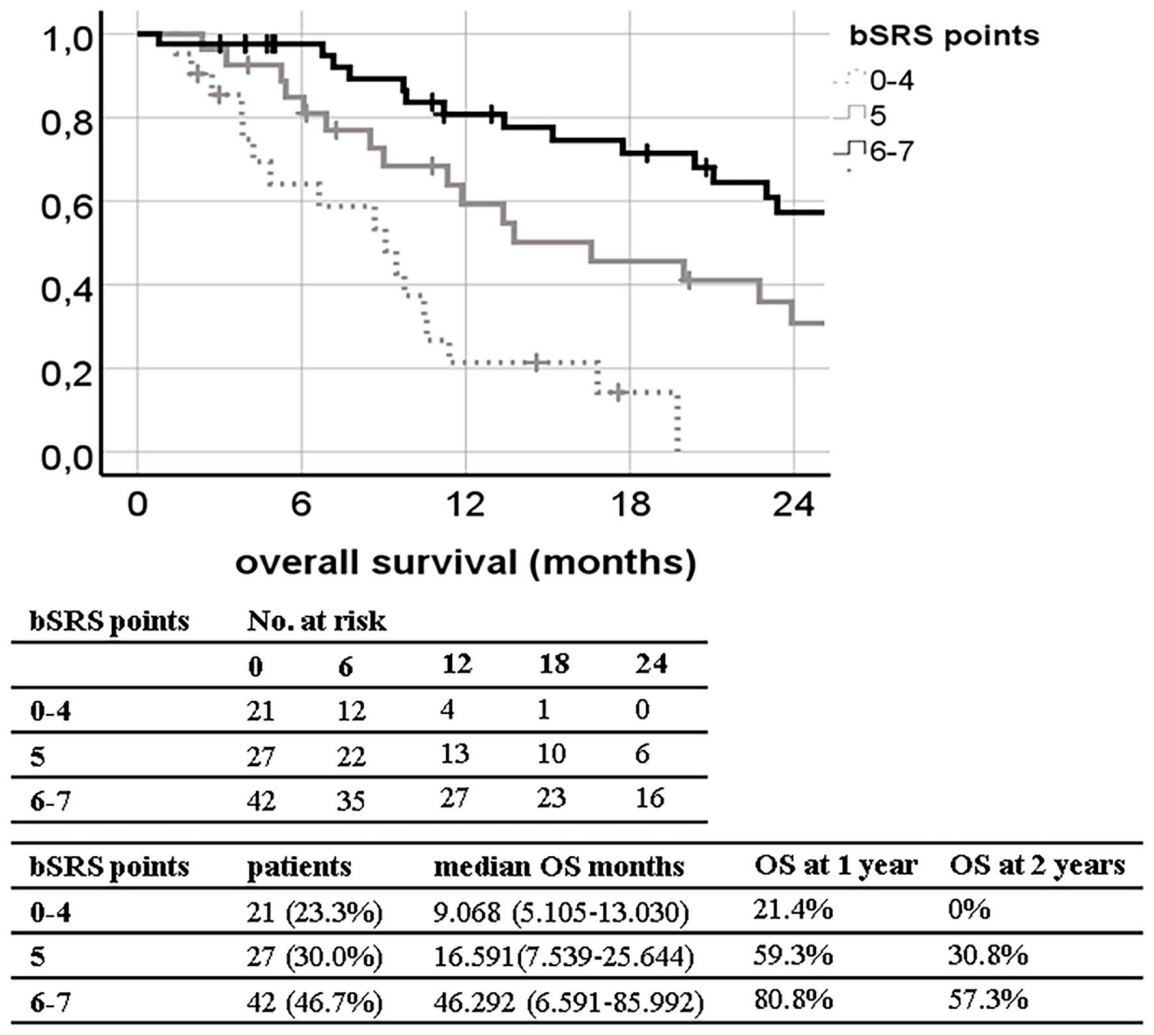
Kaplan–Meier curves for overall survival (OS; *n* = 90) divided by the breast scoring tool for stereotactic therapy (bSRS) in our patient population (log-rank *p* < 0.001; concordance index 0.689; 95% CI, 0.577–0.802; *p* = 0.003) with 1- and 2-year OS estimates.

**Figure 4:**
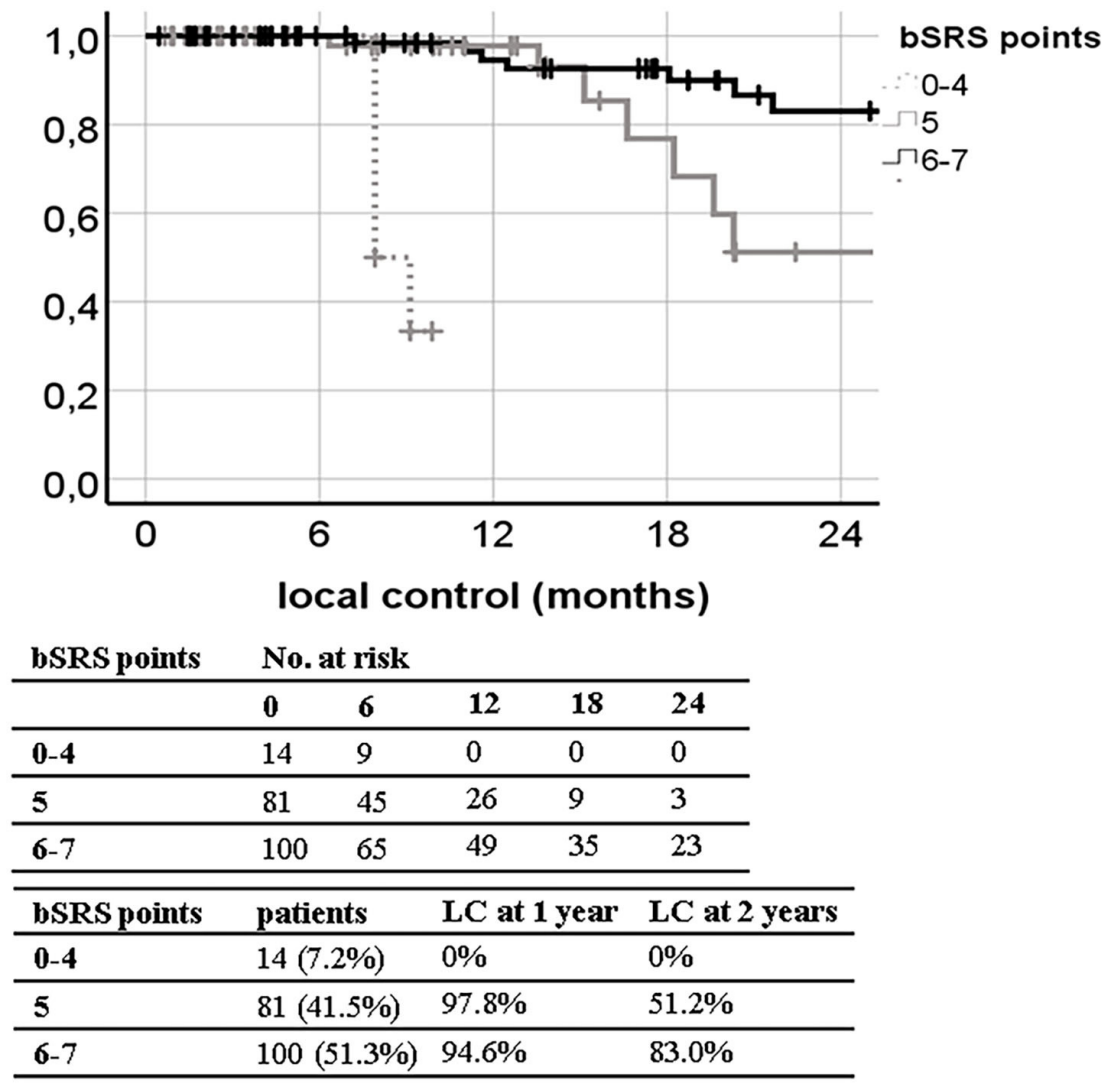
Kaplan–Meier curves for local control (LC; *n* = 195) of the irradiated lesions (*n* = 195) divided by the breast scoring tool for stereotactic therapy (bSRS) in our patient population (log-rank *p* < 0.001; concordance index 0.607; 95% CI, 0.462–0.751; *p* = 0.118) with 1- and 2-year LC estimates.

**Figure 5:**
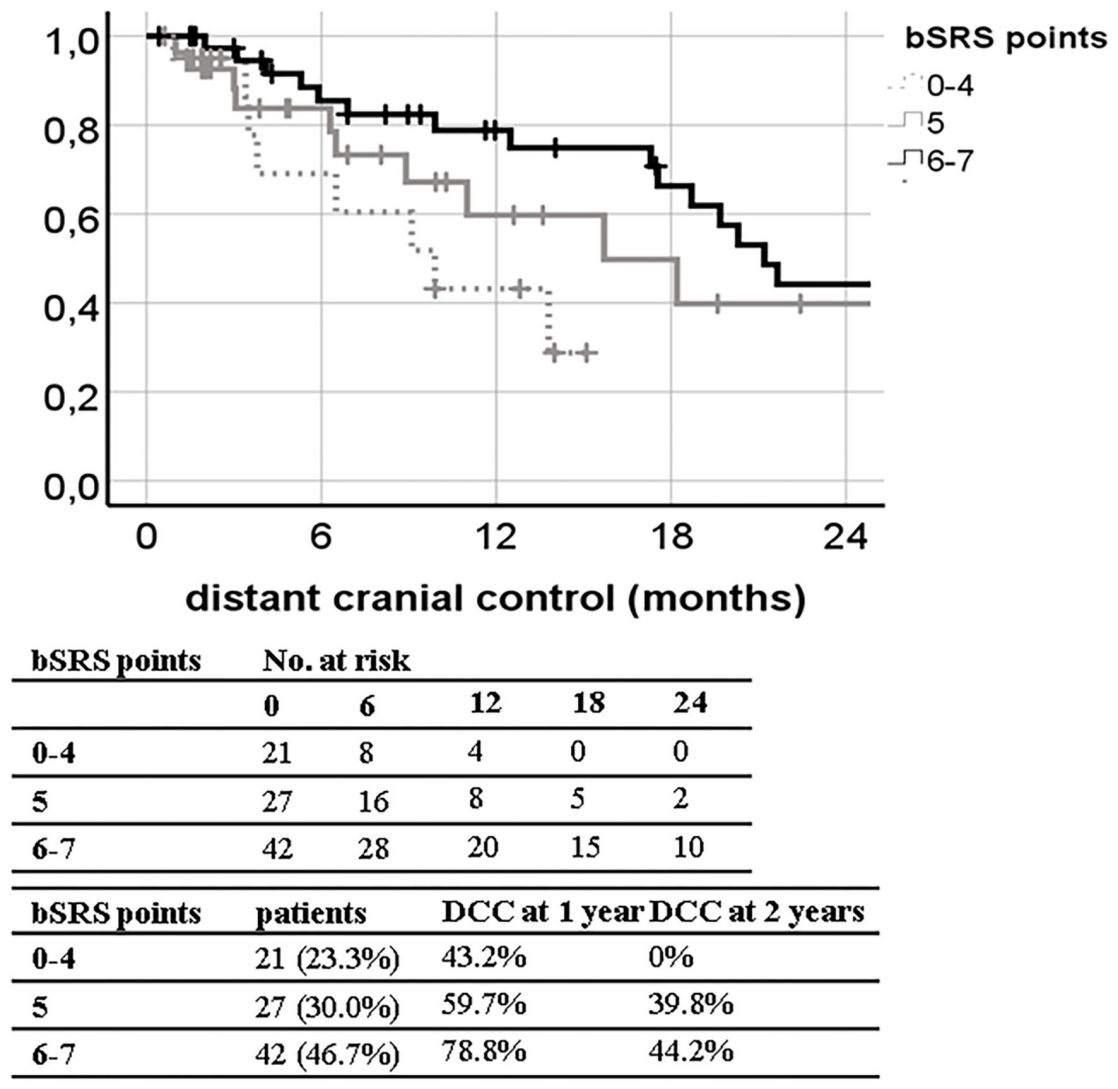
Kaplan–Meier curves for distant cranial control (DCC; *n* = 90) divided by the breast scoring tool for stereotactic therapy (bSRS) in our patient population (log-rank *p* = 0.036; concordance index 0.482; 95% CI, 0.359–0.604; *p* = 0.771) with 1- and 2-year DCC estimates.

**Figure 6:**
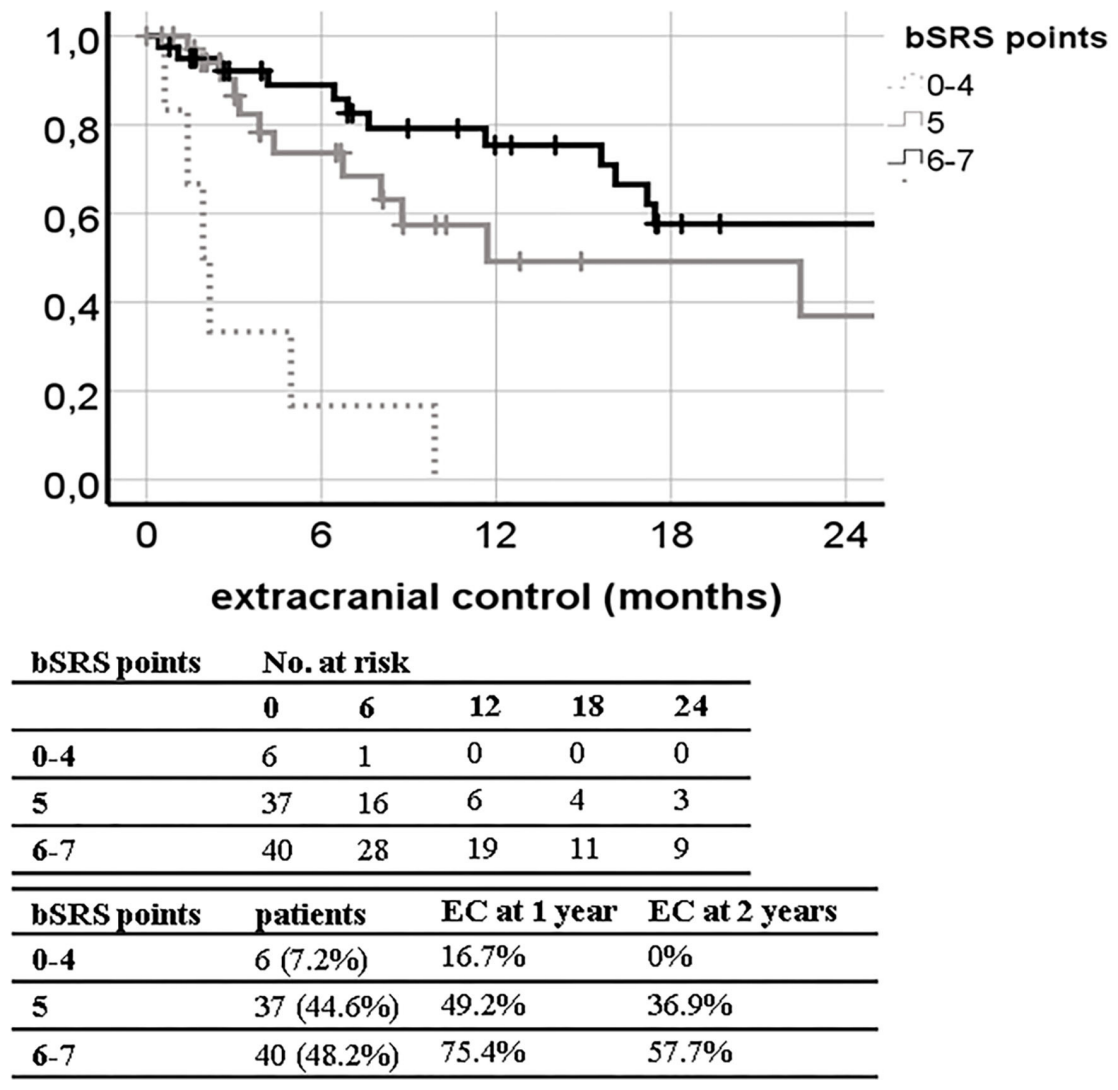
Kaplan–Meier curves for extracranial control (EC; *n* = 83) divided by the breast scoring tool for stereotactic therapy (bSRS) in our patient population (log-rank *p* < 0.001; concordance index 0.596; 95% CI, 0.465–0.728; *p* = 0.143) with 1- and 2-year EC estimates.

The bSRS did significantly classify patients into three different prognostic subgroups for all outcome variables including LC, DCC, EC, and OS. The c-index was significant for OS (0.689; 95% CI, 0.577–0.802; *p* = 0.003), but not for LC, DCC, and EC. The c-index of the bSRS was superior to those of the bGPA or the mod-bGPA (0.689 vs. 0.631 vs. 0.662). The Kaplan–Meier curves and respective OS, LC, DCC, and EC estimates are shown in Figures 3–6.

## Discussion

In this retrospective study consisting of 95 breast cancer patients who received SRS for in total 203 brain metastases, two out of nine prognostic scoring indexes could be validated for OS assessment. Furthermore, we generated a new scoring index based on prior identified prognostic factors, which was superior for OS prognosis and was also applicable for LC, DCC, and EC assessment following radiosurgery.

Our study population matches with other studies on SRS for brain metastases in breast cancer patients in terms of epidemiology, radiotherapy protocols, LC, DCC, and OS (Table 7). The tendency of higher OS in the most recent studies suggests improved systemic therapy (6, 15).

**Table 7:** Larger recent studies on radiosurgery only for brain metastases in female breast cancer patients.

	**Patients, design, characteristics**	**Treated brain metastases**	**Prescribed dose**	**Significant prognostic factors in multivariate analysis**	**Toxicity**	**2 y. LC**	**2 y. DCC**	**2 y. EC**	**2 y. OS**
Kondziolka et al. (22)	*n* = 350 Median age: 54a KPS ≥90: 79% HR+: 50% Her2+: 60% Uncontrolled extracranial disease: 67% Symptomatic: 64% Prior WBRT: 65% Prior resection: 9%	*n* = 1,535 Median FU 7.3 m (0.4–144) 1 lesion: 53% ≥5 lesions: 22.3% Infratentorial: 28%	Median 17.0 Gy (range 8–23)	**Positive (LC):** KPS ≥70, no prior WBRT, smaller total tumor volume **Positive (DCC):** controlled extracranial disease, lower number of metastases at the time of SRS, absence of lung metastases **Positive (OS):** controlled extracranial disease, KPS score ≥70, smaller total tumor volume, absence of brainstem metastases, Her2+	11% “adverse radiation effects”	71% (1 y.)	NA	NA	26%
Yamamoto et al. (14)	*n* = 269 Median age: 55a KPS ≥80: 78% Symptomatic: 60% Prior WBRT: 4% Prior resection: 24%	*n* = NA Median FU 9.9 m (range, 0.1–122.3 months) 1 lesion: 22%	Median 21 Gy (range 10–25)	NA	NA	NA	NA	NA	21%
Yang et al. (15)	*n* = 136 Median age: 58a KPS ≥80: 89% HR+: 32% Her2+: 42% TNBC: 26% Active extracranial disease: 54% Breast GPA ≥2.5: 63%	*n* = 186 Median FU: 23.4 m (2.3–140.2) 1 lesion: 59% Max 3 lesions Infratentorial: 32%	Median dose 21 Gy (range 14–24) @ 80%	**Negative (LC)**: max. diameter of brain metastasis **Negative (DCC):** active extracranial disease, not receiving active systemic therapy **Negative (OS):** >1 lesion, TNBC, active extracranial disease	8.6% radionecrosis (defined as no tumor in histology)	71%	43%	NA	41%
Armstrong et al. (6)	*n* = 56 Median age: 53a KPS ≥80: 76% HR +: 52% Her2+: 59% Symptomatic: 61% Prior WBRT: 43%	*n* = 94 Median FU: 10.33 months (1.25–97.28) 1 lesion: 59% Max 5 lesions	12–22 Gy (1 fraction); 24 Gy (3 fractions); 30 Gy (6 fractions)	**Negative (OS):** uncontrolled extracranial disease at the time of SRS	No grade III toxicity	NA	NA	NA	36%
Weykamp et al. (present study)	*n* = 95 Median age: 57 KPS ≥80:73% HR+: 59% Her2+:38% TNBC: 28% Symptomatic: 34% Extracranial progression: 6% Prior WBRT: 32% Prior resection: 18% Breast GPA ≥2.5:55%	*n* = 203 Median FU 11.9 m (0.8–105.3) 1 lesion: 51.6% Max 7 lesions Infratentorial: 21.4%	18–20 Gy (1 fraction); 30 Gy (6 fractions) @ 70–90%	**Positive (LC):** time to metastases ≥18 months **Negative (LC):** irradiated lesion is symptomatic, extracerebral progression **Positive (DCC):** KPS ≥80% **Negative (EC):** extracerebral progression **Positive (OS):** KPS ≥80%, Her2+	5.3% radiation induced changes (2 out of 5 proven by surgery)	72%	37%	47%	38%

*a, year; DCC, distant cranial control; EC, extracranial control; GPA, graded prognostic assessment; FU, follow-up; HR, hormone receptor; KPS, Karnofsky Performance Status; LC, local control; m, months; NA, not available; OS, overall survival; SRS, stereotactic radiosurgery; TNBC, triple-negative breast cancer; WBRT, whole-brain radiotherapy*.

Seven out of nine examined, well-established scoring indexes for radiotherapy of brain metastasis patients failed to yield significantly different prognostic groups in our study population, which may be caused by the different composition of the respective prognostic scoring indexes.

The PSS could not separate our patient cohort in different prognostic subgroups, although it was specifically designed for breast cancer patients with brain metastases receiving SRS (15). Nonetheless, it does not include the KPS (Table 1), even though this was reported to be one of the most important prognostic factors as shown previously (4, 5) and in our analysis. Thus, the KPS was also included in our bSRS. The mRPA, which also showed no prognostic relevance in our study cohort, was as well specifically designed for breast cancer patients with brain metastases receiving SRS. However, it is only a modification of the old RPA and therefore does not include the hormone or Her2/neu receptor status (14), which might explain the inferior results.

Only the bGPA and the mod-bGPA were confirmed as reliable prognostic tools in our study population as measured by the c-index (Figures 1, 2). A major reason for this finding might be that these two prognostic tools take into account the histological peculiarity of breast cancer patients, especially the Her2/neu receptor status (4, 5). Several studies have confirmed an independent positive impact of Her2/neu receptor positivity on OS in breast cancer patients after radiation of brain metastases (15, 22, 23). This was also demonstrated in our multivariate analysis (Table 2), which led to the inclusion of the Her2/neu receptor status into our bSRS. One of the reasons why Her2/neu receptor positivity improves OS is thought to be the anti Her2/neu therapy agents acting as a radiosensitizer (24).

In our analysis, both bGPA and mod-bGPA have their weakness in discriminating the second and third most favorable prognostic groups during the first 24 months of follow-up as illustrated in Figures 1, 2. This is probably caused by the fact that especially the most unfavorable prognostic group of the mod-bGPA is unproportionally small (*n* = 3; 3%; Figure 2) compared to the respective validation study by Subbiah et al. (5) (21%). However, this can be explained by the mod-bGPA being predominantly validated for WBRT. Only 11% of the validation study population was to receive SRS alone, since 42% of the patients had more than five brain metastases (5). In our SRS-only study population, 88% patients had a maximum of three brain metastases. Consequently, attributing a higher score to patients with only up to three brain metastases, like the mod-bGPA, grants artificial scoring points to nearly the entire study population. This results into a smaller subgroup of patients with only few total scoring points. Nonetheless, the mod-bGPA yields a higher c-index, indicating to be more suitable for differentiating prognostic OS subgroups than the bGPA (Figures 1, 2). The mod-bGPA includes a modified scoring system for age discrimination. Patients with older age are less disadvantaged than with the bGPA. This might improve prognostic reliability considering that older age could not be identified as an independent risk factor for inferior survival, neither in the present study nor in the recent comparable studies (6, 15, 22).

In 2014, Yamamoto et al. demonstrated that OS was non-inferior for patients treated with radiosurgery for 5–10 brain metastases compared to those who received SRS for only 2–4 cerebral metastases (25). This led to a change in treatment practice in the radiooncology community with an increasing number of centers offering radiosurgery for more than 4 brain metastases for selected patients. Hence, today the prognostic quality of several of the investigated scoring indexes, which are at least partly based on the number of brain metastases, like the mod-bGPA, is impaired. These indexes do no longer reflect current treatment approaches emphasizing again the necessity for a modern prognostic score. It remains unclear why the u-bGPA could not be successfully validated in our study population although being the latest bGPA scoring tool to date (12). Possibly, this is due to the fact that the prognostic factor “presence of extracranial metastases” was reintegrated into the u-bGPA (Table 1), like it was originally used in the old, not breast cancer-specific GPA (3). However, for the u-bGPA, Sperduto et al. only used the sole status of extracranial metastases without any quantitative measurement of the metastatic burden (e.g., dissemination) (12). Furthermore, no information on extracranial disease progression was provided, although it was described as an important independent factor for OS prognosis and is therefore also part of our bSRS (6, 15, 22). Since systemic therapies in breast cancer have improved during the last years, but often still do not penetrate the blood–brain barrier or even increase the risk of radiation-induced changes when combined with SRS, extracranial progression is a highly conflicting circumstance (26, 27). In general, patients with controlled extracranial disease may benefit the most from upfront definitive SRS alone, as shown in our study. However, in patients with progressive extracranial disease, SRS might offer the benefit of minimal systemic treatment interruption compared to WBRT and hereby enabling medical oncologists to continuously pursue optimal extracranial disease control with systemic treatments.

Our developed bSRS index consists of well-known prognostic factors for contemporary patients including the KPS, the Her2/neu status, and the status of extracranial progression as discussed above. The bSRS index could successfully divide patients into three significantly different subgroups in terms of LC, DCC, EC, and OS altogether. However, the c-index was only found to be significant for OS, and not for LC, DCC, and EC. This might be due to the main limitations of the presented study, namely, its retrospective and single-center design, leading to a rather small sample size. Furthermore, extracranial progression was much less frequent in the present study (6%) compared to other studies in the field with up to 67% (15, 22). This circumstance reflects a more conservative utilization of SRS in our patient cohort and thus hinders the development of an all-encompassing prognostic index, since extracranial progression was the only independent factor, both negatively influencing LC and EC to be identified (Table 2). Nonetheless, the bSRS was superior in differentiating prognostic OS subgroups compared to all other indexes as indicated by the higher c-index and the properly separated Kaplan–Meier curves (Figures 1–3), since it reflects the latest knowledge of prognostic OS factors in treatment of limited brain metastases in breast cancer patients, namely, KPS, Her2/neu positivity, and extracranial control (Tables 1, 7).

## Conclusion

This retrospective single-center study could validate the bGPA and mod-bGPA as prognostic tools for OS in 95 breast cancer patients receiving SRS for in total 203 brain metastases. Furthermore, we developed the bSRS, a modern risk score for assessing not only OS but also LC, DCC, and EC following radiosurgery for breast cancer patients. Larger studies or *post-hoc* analyses of trials with a higher proportion of extracranial progressive patients are required to externally validate our generated score index.

## Data Availability Statement

The datasets generated for this study are available on request to the corresponding author.

## Ethics Statement

The studies involving human participants were reviewed and approved by Heidelberg University ethics committee on April 12th, 2018 (#S-172/2018). Written informed consent for participation was not required for this study in accordance with the national legislation and the institutional requirements.

## Author Contributions

FW carried out the data collection, performed the statistical analysis, and drafted the manuscript. RE, LK, KS, TF, PH, SR, and NA helped with data collection as well as figure and table preparation. RE, FW, LK, KS, NA, and JH-R were involved in patient treatment. TD and AS contributed to the gynecological knowledge of the manuscript and were involved in pre-radiotherapy treatment. JH-R and JD participated in the study design and helped to draft the manuscript. All the authors were responsible for data interpretation, participated in manuscript revisions, and approved the final manuscript.

## Conflict of Interest

The authors declare that the research was conducted in the absence of any commercial or financial relationships that could be construed as a potential conflict of interest.
